# Quantitative Comparison of Mathematical Models to Measure Surface Area of Canine Teeth Prepared to Receive Full Veneer Crowns in Dogs

**DOI:** 10.3389/fvets.2015.00031

**Published:** 2015-09-07

**Authors:** Caitlyn J. Collins, Scott Joseph Hetzel, Sarah Siverling, Heidi-Lynn Ploeg, Jason W. Soukup

**Affiliations:** ^1^Bone and Joint Biomechanics Laboratory, Department of Mechanical Engineering, University of Wisconsin-Madison, Madison, WI, USA; ^2^Department of Biostatistics and Medical Informatics, University of Wisconsin-Madison, Madison, WI, USA; ^3^Dentistry and Oral Surgery, School of Veterinary Medicine, University of Wisconsin-Madison, Madison, WI, USA

**Keywords:** crown surface area, full veneer crown, prosthodontics, crown preparation, tooth preparation, crown preparation design, mathematical models, dogs

## Abstract

**Objective:**

This study was performed in order to determine if mathematical modeling of the canine teeth in dogs could be utilized to provide an accurate and reliable estimation of crown surface area that could be used in both a research and a clinical setting.

**Materials and methods:**

Actual surface area (aSA) calculations for 32 stone dies of clinical crown preparations were acquired utilizing a tridimensional (3D) laser scanner and 3D computer-aided design and manufacturing (CAD/CAM) software applications. These calculations were used as a control. Seventeen unique mathematical models from eight geometric shapes were used to calculate estimated surface area (eSA) of each stone die. Linear association and agreement between eSA and aSA calculations were assessed with multiple statistical methods.

**Results:**

All methods of eSA showed a significant linear association with aSA. Five of the mathematical models [right elliptical frustum (H3), right elliptical cone (G3), right pyramidal cone (A3), right circular frustum (F2), and right circular cone (E1)] were superior to the other 12 models.

**Conclusion:**

The H3 mathematical model based on the right elliptical frustum provided the most accurate estimate of crown surface area of dog teeth. However, H3 requires the use of laser scans and a 3D CAD software program. As a result, this model would be recommended for research applications. The E1 mathematical model was similar in accuracy to H3 and, given it requires only two measurements and a comparatively simple equation for calculation, this method would be recommended for clinical chair-side use.

## Introduction

There are several principles pertaining to tooth preparation that contribute to the long-term success of a full veneer crown. One of these principles is the consideration of retention and resistance form (1). There are few features, which contribute to retention and resistance form that are under operator control. However, the operator has some control over the geometric configuration of the preparation, including tooth height, diameter, convergence angle, and auxiliary features. Studies on human and dog teeth have shown that for a given base diameter and convergence angle, the greater the height of a tooth the more likely it is to have retention and resistance form. Similarly, for a given base diameter and height, a greater convergence angle leads to a lower likelihood of obtaining sufficient retention and resistance form (2, 3).

Operator manipulation of crown height, base diameter, and convergence angle as well as the addition of auxiliary features will have an influence on crown surface area (SA). Kaufman et al. evaluated the influence of various preparation design features on the retention of full veneer crowns and demonstrated a positive correlation between SA and unseating force (4). This positive association has subsequently been confirmed in dogs (5).

Most studies evaluating the influence of tooth preparation design on retention of cast restorations either minimize or completely ignore the influence of SA. This is likely due to the fact that most of the foundational studies determining what constitutes good resistance and retention form were performed prior to the development of modern luting cements that have very high bonding strength (6, 7). Due to the poor bonding strength of early luting cements, such as zinc phosphate, described principles of tooth preparation depend primarily on the geometrical design of the preparation (4).

Zinc phosphate cements are generally no longer utilized due to their poor bonding properties compared to more modern cements. El-Mowafy et al. determined that the amount of force required to dislodge a crown cemented onto a die with poor resistance/retention features was increased 3.5-fold when resin-based cements were utilized instead of zinc phosphate (7). Soukup et al. also suggested that the use of resin-based cements in dogs allows the use of large convergence angles (8). Therefore, the use of modern cements reduces the dependence on opposing axial wall parallelism and increases the usefulness of methods to increase SA.

A preparation’s SA is largely dictated, but not necessarily restricted, by the physical dimensions of the preparation. The operator does maintain some control of a preparation’s SA. Adding surface features such as grooves and boxes to the preparation, utilizing subgingival margins (although not advocated by the authors), or performing surgical crown lengthening are methods to increase preparation SA (9).

An accurate and clinically applicable method of measuring a tooth preparation’s SA would be a useful tool for researchers and clinicians. Because of the irregular shape of a tooth, the exposed SA cannot be measured without a reverse engineering tool, such as a laser scanner (10). We have previously shown that a laser scanner can be utilized to acquire SA measurements of canine teeth in dogs (5). However, laser scanning is time consuming, expensive, and not practical for chair-side use or for clinical research. In order to more easily acquire an accurate chair-side SA measurement of a tooth, a simpler methodology is necessary.

The aim of the present study was to determine if there is a mathematical model using geometric shapes that, when using tooth dimensions acquired from clinical cases, can accurately model and predict the SA of a prepared canine tooth.

## Materials and Methods

Stone dies of 32 maxillary and mandibular canine teeth from clinical patients of the University of Wisconsin-Madison Veterinary Care Dentistry and Oral Surgery Service were utilized for the present study. The dies were from single-unit full veneer preparations that occurred between the years of 2002 and 2008.

**Figure 1:**
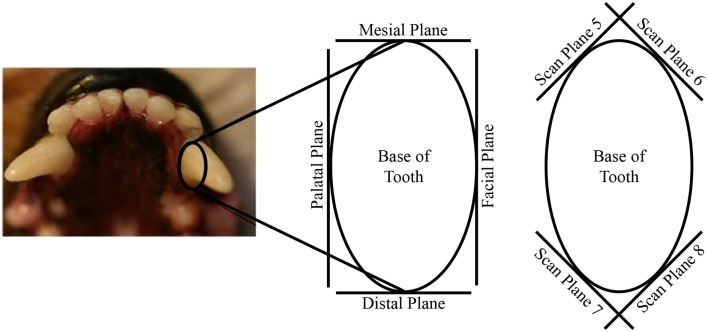
**Illustration depicting the 3D laser scan planes of the tooth dies**.

The tooth dies were scanned using a laser scanner (ShapeGrabber LM600, Shape Grabber, Inc., Ottawa, ON, Canada). Each tooth die was scanned in 10 specific orientations in order to capture the entire exposed tooth crown SA and gingival preparation margin (GPM). The 10 orientations used to scan each tooth die were comprised of the four axial planes of each tooth (facial, mesial, palatal, and distal), the four planes at a 45° angle from intersecting axial planes, and the top and bottom (Figure 1).

**Figure 2:**
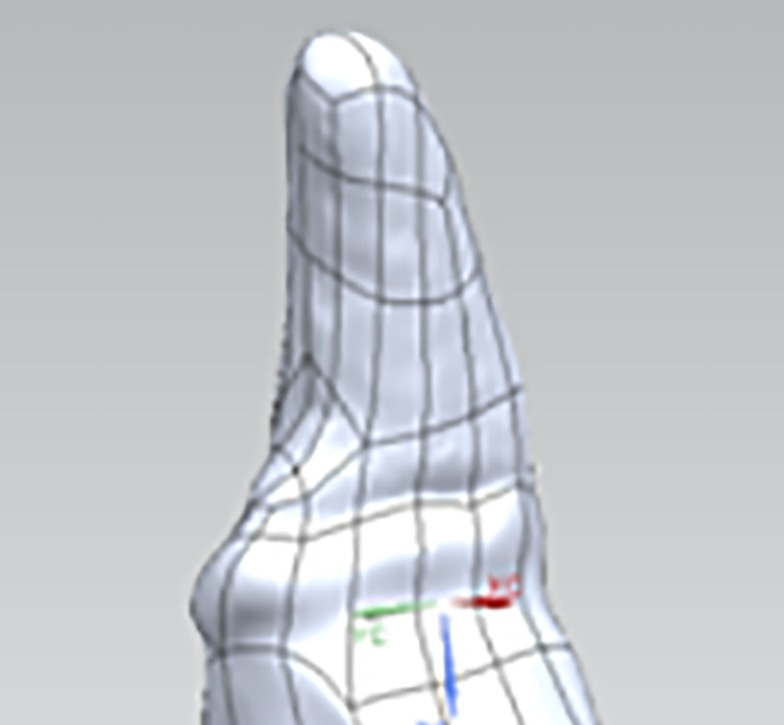
**Representative 3D model of a tooth die with NURBS surfaces**.

The 3D surface coordinates collected from the laser scanner were imported into a reverse engineering and 3D modeling software (Geomagic Studio 2011, Geomagic, Research Triangle Park, NC, USA). The point clouds made up of the 3D surface coordinates from each of the scans were aligned, wrapped, and merged to form a 3D model of the tooth. The 3D models were used to create non-uniform rational B-spline (NURBS) surfaces (Figure 2). The edges of the NURBS surfaces near the tooth were adjusted to follow the GPM. The NURBS surfaces were then saved as the computer-aided design (CAD) geometry format Initial Graphics Exchange Specification (IGES).

The IGES files of each tooth were then imported into a 3D CAD software program (Solidworks 2011, Dassault Systèmes, Waltham, MA, USA) and the exposed tooth SA, or actual SA (aSA), was calculated after all the NURBS surfaces of the tooth coronal to the GPM were selected. The aSA served as the control.

**Figure 3:**
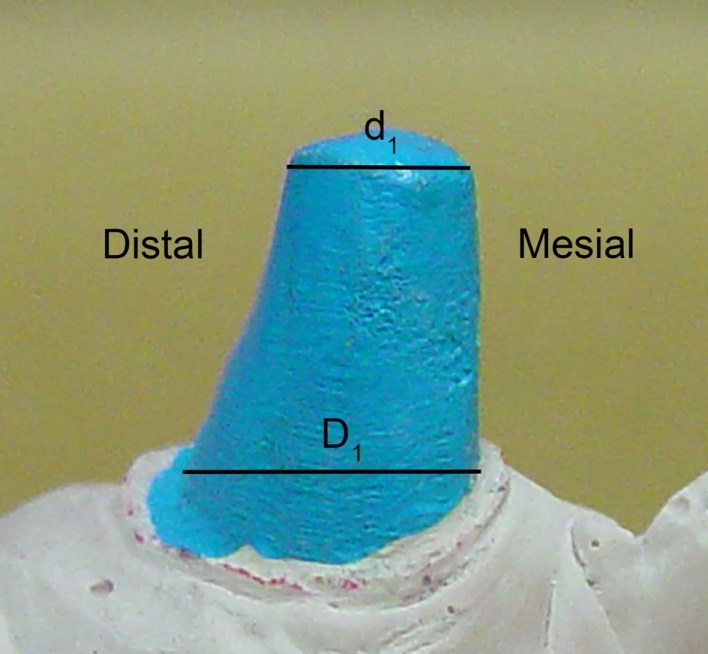
**Figure depicting the tooth die measurements (*D*_1_ and *d*_1_) used for the eSA calculations**.

**Figure 4:**
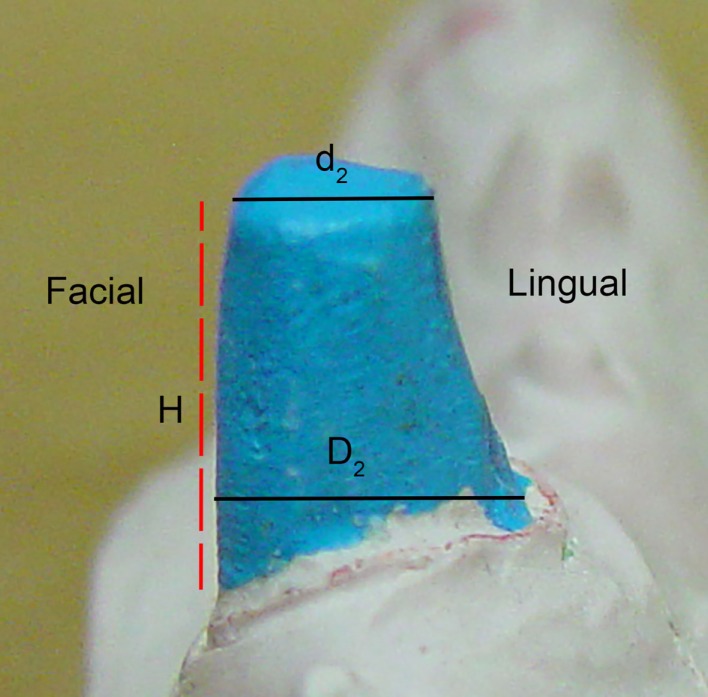
**Figure depicting the tooth die measurements (*D*_2_, *d*_2_, and *H*) used for the eSA calculations**.

The IGES files of each tooth were then saved as Parasolid Binary images and imported into a computer-aided design, manufacturing, and engineering (CAD/CAM/CAE) software program (NX 7.5, Siemens, Munich, Germany) where five dimensions were measured on each tooth using standardized clinical procedures: height (*H*), base major axis (*D*_1_), base minor axis (*D*_2_), top mesial–distal axis (*d*_1_), and facial–palatal axis (*d*_2_) (Figures 3 and 4). The measured tooth dimensions were utilized in various combinations to yield 17 unique estimated tooth SA (eSA) calculations from eight basic geometric shapes as detailed in Table 1. The base of each geometric shape was excluded from the SA calculations since the measured SA of each tooth was restricted to the exposed SA.

**Table 1:** **Geometric shape variations calculated for each tooth**.

Shape	Illustration of geometric shape	Surface area calculation	Variations
Right pyramidal cone	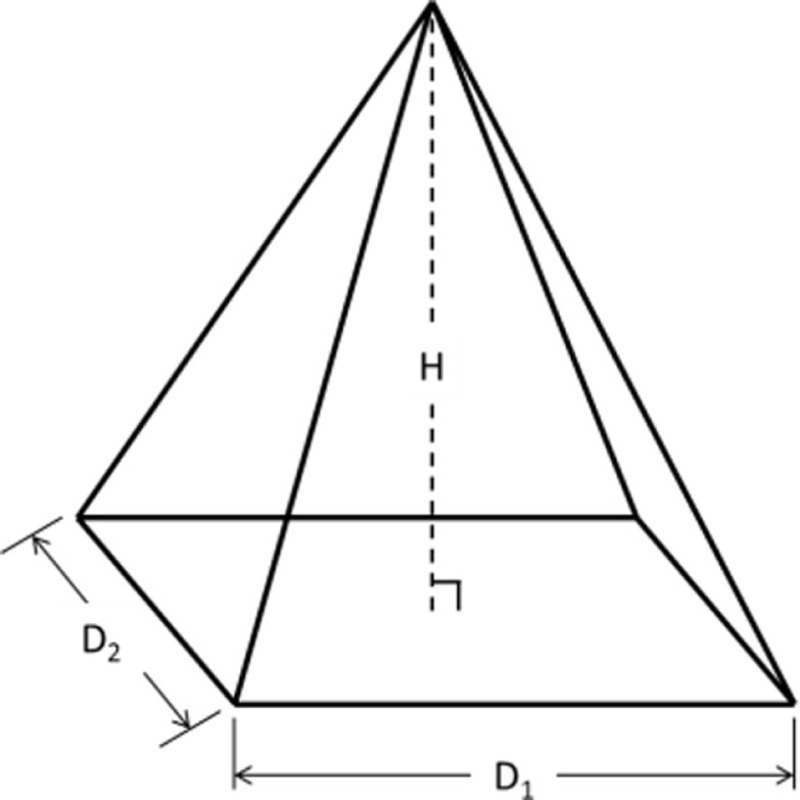	$D_{1}\sqrt{H^{2}+\frac{{D_{2}}^{2}}{4}}+D_{2}\sqrt{H^{2}+\frac{{D_{1}}^{2}}{4}}$	A1: *D*_1_ = *D*_2_ = base major dimension (MaD) A2: *D*_1_ = *D*_2_ = base minor dimension (MiD) A3: *D*_1_ = base MaD, *D*_2_ = base MiD
Right pyramidal frustum	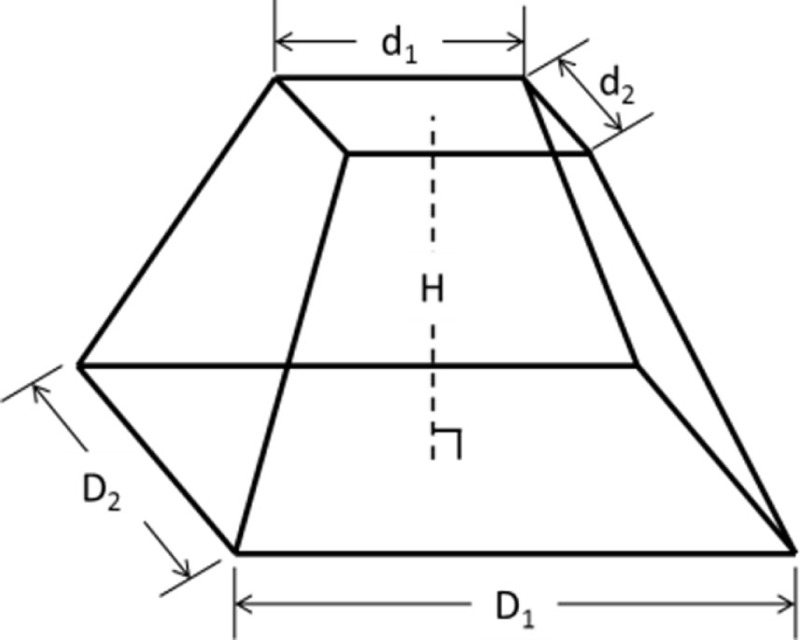	$d_{1}d_{2}+\left(D_{1}+d_{1}\right)\sqrt{H^{2}+\frac{1}{4}{\left(D_{2}-d_{2}\right)}^{2}}$ $+\left(D_{2}+d_{2}\right)\sqrt{H^{2}+\frac{1}{4}{\left(D_{1}-d_{1}\right)}^{2}}$	B1: *D*_1_ = *D*_2_ = base MaD and *d*_1_ = *d*_2_ = top mesial–distal dimension (MD) B2: *D*_1_ = *D*_2_ = base MiD and *d*_1_ = *d*_2_ = top facial–palatal dimension (FP) B3: *D*_1_ = base MaD, *d*_1_ = top MD, *D*_2_ = base MiD, and *d*_2_ = top FP
Right cuboid	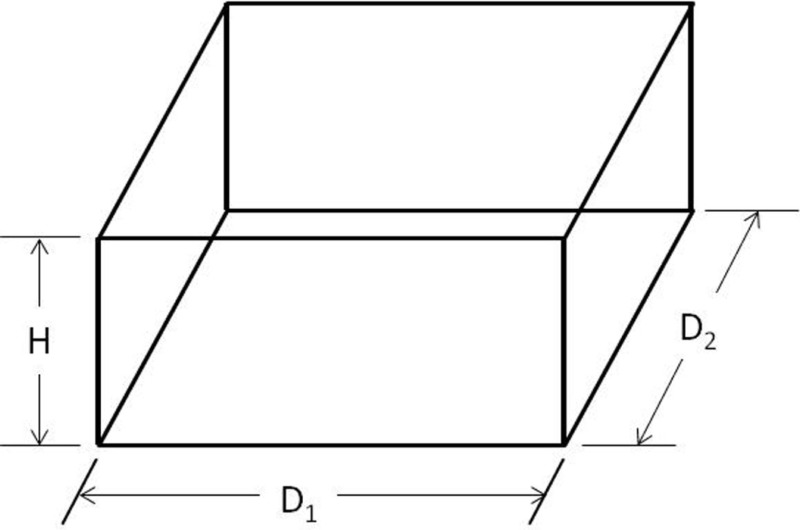	*D*_1_*D*_2_ + 2*H*(*D*_1_ + *D*_2_)	C1: *D*_1_ = *D*_2_ = base MaD C2: *D*_1_ = *D*_2_ = base MiD C3: *D*_1_ = base MaD and *D*_2_ = base MiD
Right cylinder	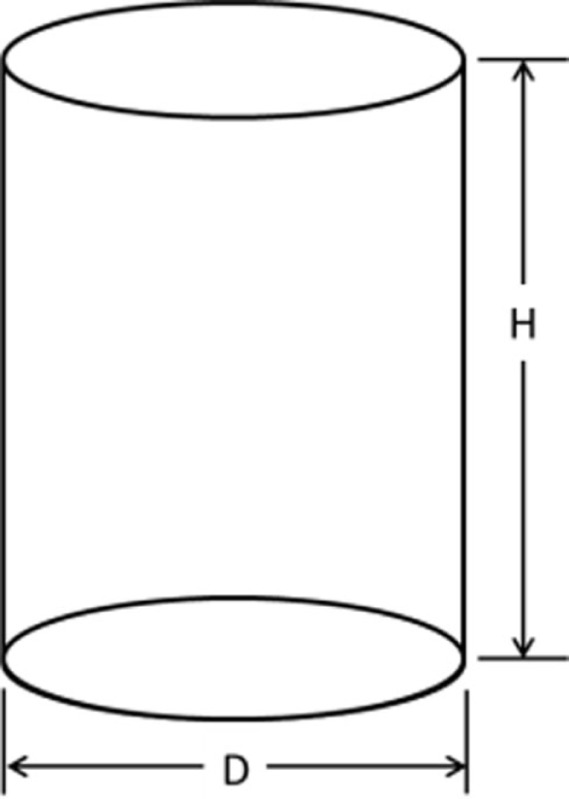	$\pi D\left(\frac{D}{4}+H\right)$	D1: *D* = base MaD D2: *D* = base MiD
Right circular cone	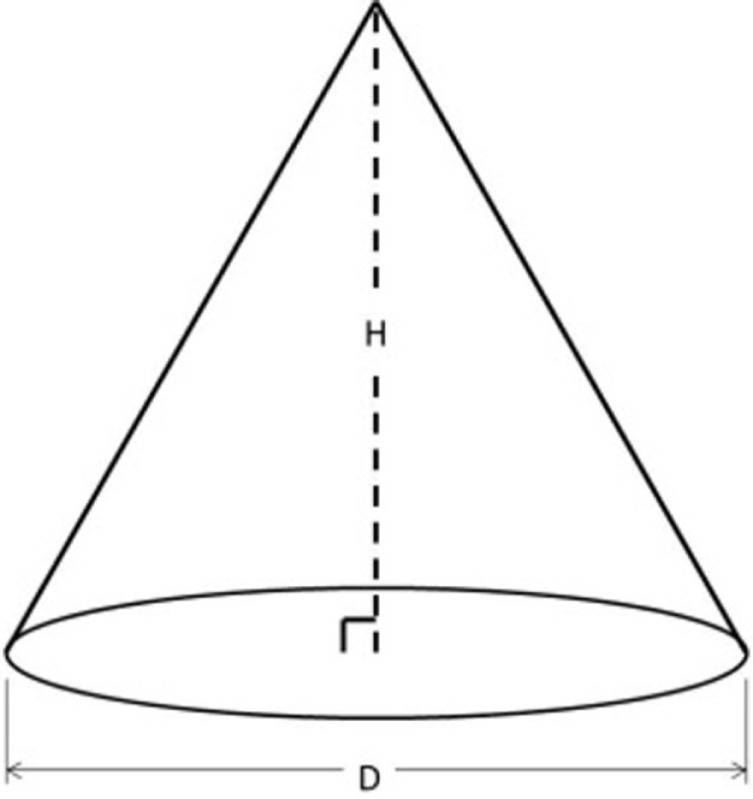	$\frac{\pi D}{2}\sqrt{H^{2}+\frac{D^{2}}{4}}$	E1: *D* = base MaD E2: *D* = base MiD
Right circular frustum	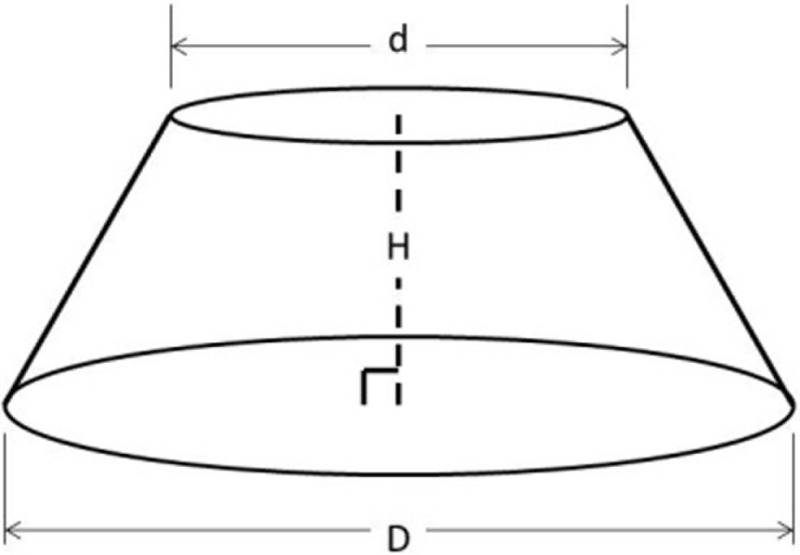	$\pi \left(\frac{D}{2}+\frac{d}{2}\right)\sqrt{D^{2}-2\mathrm{Dd}+d^{2}+4H^{2}}+\frac{\pi d^{2}}{4}$	F1: *D* = base MaD and *d* = top MD F2: *D* = base MiD and *d* = top FP
Right elliptical cone	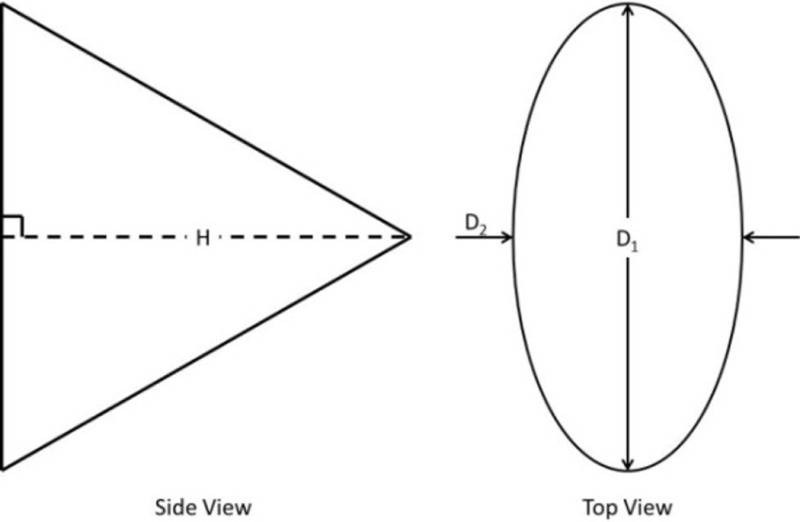	Solved in Maple (version 15, Maplesoft, Waterloo, ON, Canada) $D_{1}\sqrt{{\left(\frac{D_{2}}{2}\right)}^{2}+H^{2}}\mathrm{EllipticE}\left(\sqrt{\frac{1-\frac{{D_{2}}^{2}}{{D_{1}}^{2}}}{1+\frac{{\left(\frac{D_{2}}{2}\right)}^{2}}{H^{2}}}}\right)$	G3: *D*_1_ = base MaD and *D*_2_ = base MiD
Right elliptical frustum	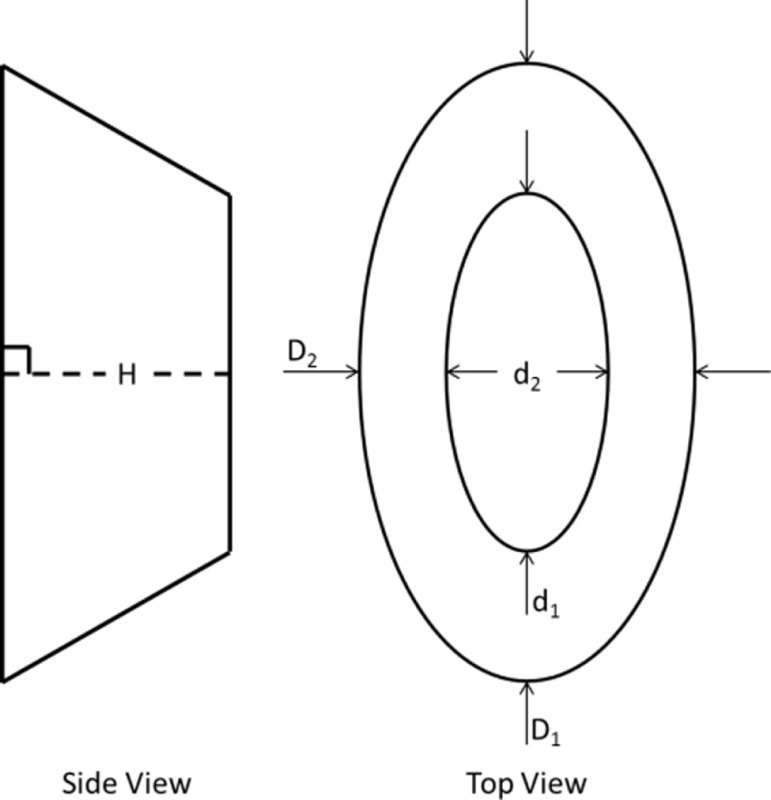	Calculated in Solidworks	H3: *D*_1_ = base MaD, *d*_1_ = top MD, *D*_1_ = base MiD, and *d*_1_ = top FP

### Statistical methods

Linear association and agreement between aSA and eSA calculations were assessed with multiple different statistical methods. Linear association of eSA with aSA was assessed with goodness of fit assessments with the Akaike information criterion (AIC) and corresponding Chi-square test for each eSA. We also determined the Pearson correlation coefficients (PCC) for each eSA with aSA. Agreement between the two methods was assessed with intraclass correlation coefficients [ICC (2,1)] as described by Shrout (11). We also assessed agreement with paired *t*-tests between aSA and each eSA method. Lastly, we assessed agreement by testing if the *y*-intercept and slope of the simple linear regression models of aSA by each eSA were significantly different than 0 mm^2^ and 1 mm^2^/mm^2^, respectively. If there is perfect agreement between aSA and any eSA, then the *y*-intercept should be 0 and the slope should be equal to 1 mm^2^/mm^2^. After choosing the best 5 eSA methods from the previously described analyses, we calculated the prediction 95% confidence interval (CI) for aSA if we had a new calculated eSA for each method.

## Results

**Figure 5:**
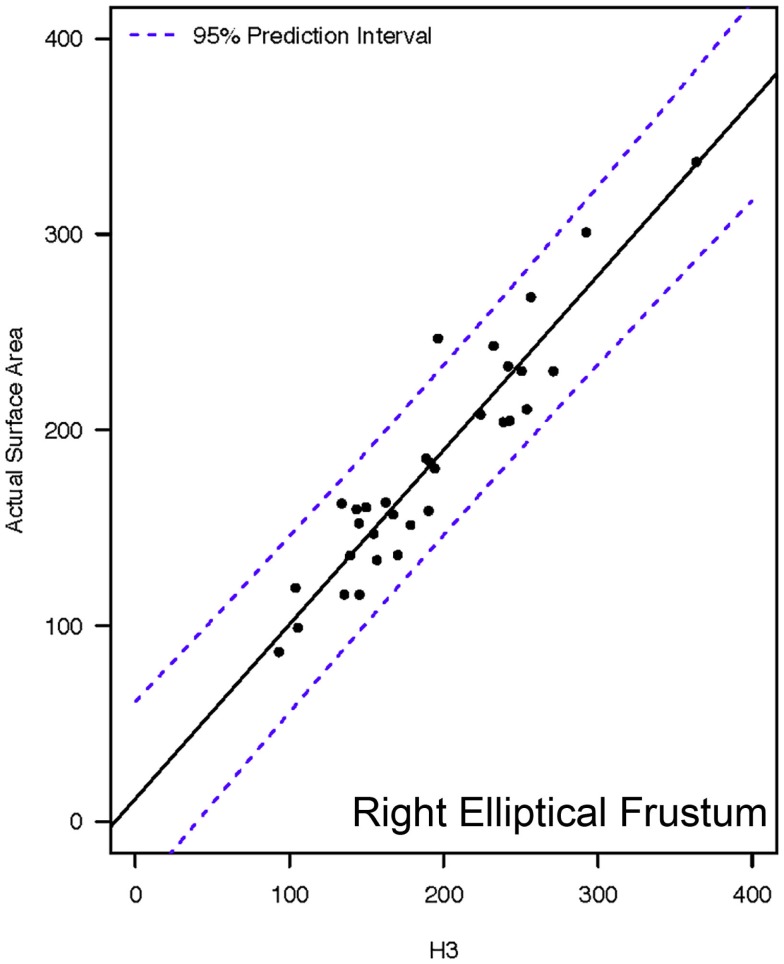
**Representative linear regression for the H3 mathematical model**. Units for the *x*- and *y*-axes are in square millimeter.

**Figure 6:**
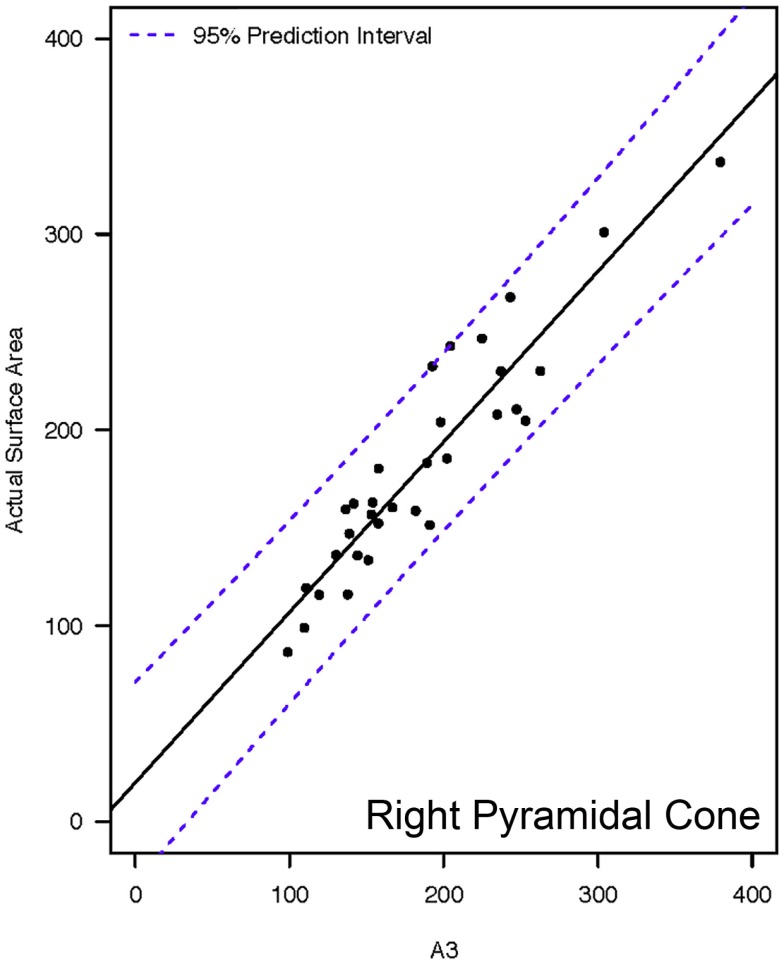
**Representative linear regressions for the A3 mathematical model**. Units for the *x*- and *y*-axes are in square millimeter.

**Figure 7:**
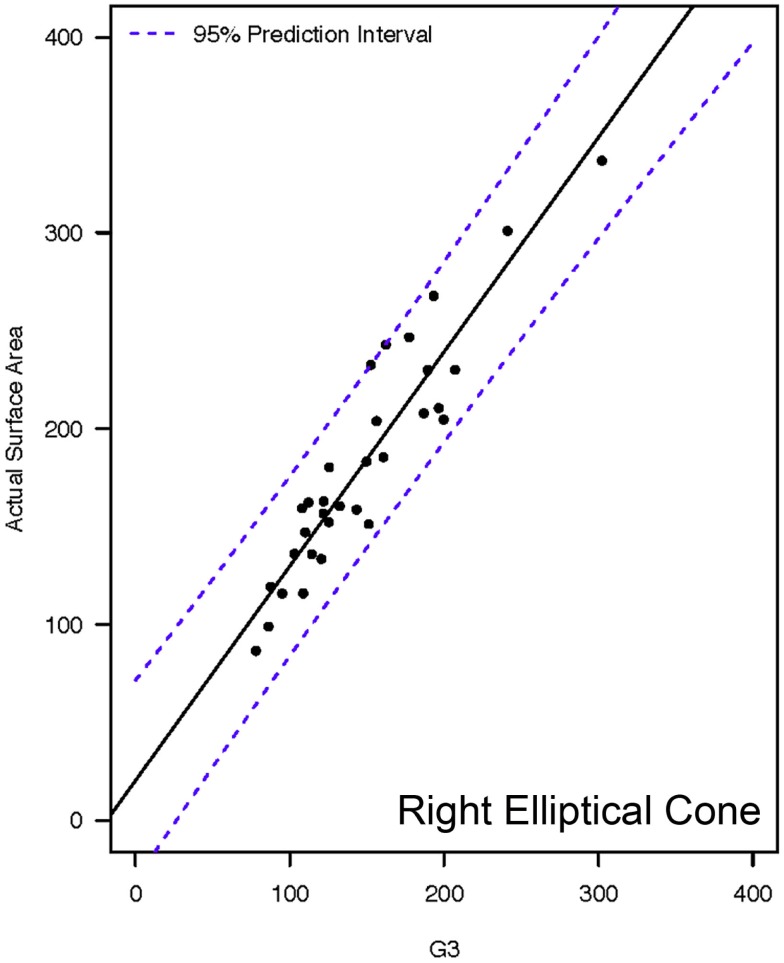
**Representative linear regressions for the G3 mathematical model**. Units for the *x*- and *y*-axes are in square millimeter.

**Figure 8:**
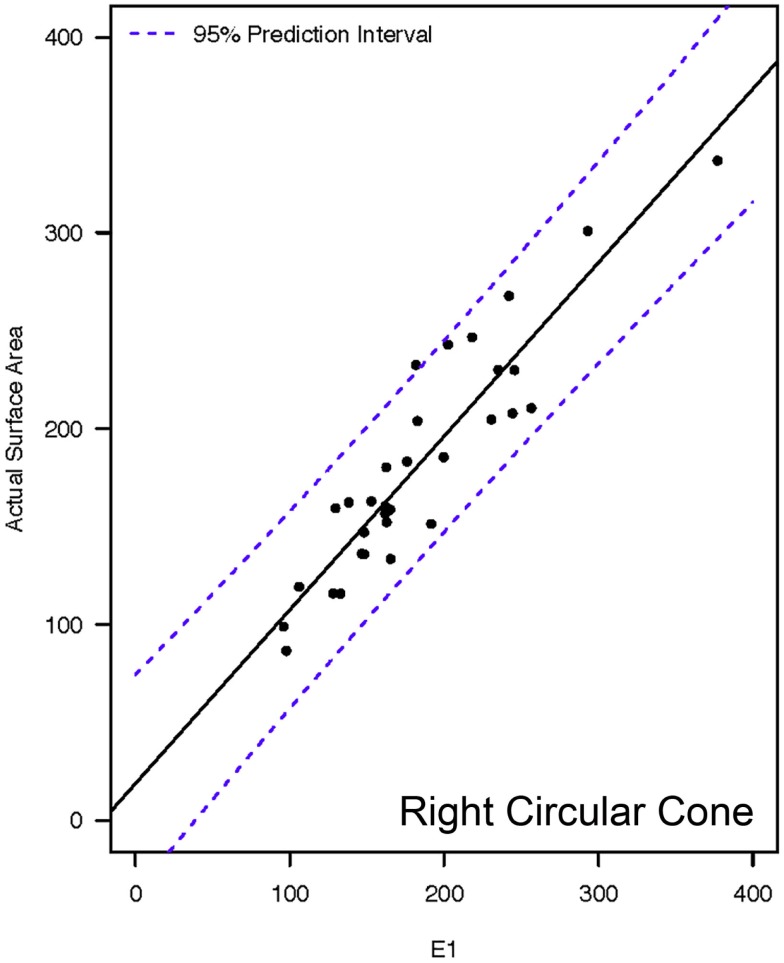
**Representative linear regressions for the E1 mathematical model**. Units for the *x*- and *y*-axes are in square millimeter.

Actual and estimated surface area calculations from the 3D laser-scanning method are provided in Table 2. All methods of eSA showed a significant linear association with aSA (all *p* < 1E−11). Methods H3 and B3 showed the best linear association and agreement with aSA with AIC values of 196.6 and 196.8, respectively, while methods G3, A3, and C3 showed the next best linear association and agreement, respectively (Table 3). PCC were all relatively high and ranged from 0.94 (H3) to 0.88 (F2 and B2) (Table 4). Agreement, as measured by ICC (2,1), varied highly across all the methods of eSA and ranged from excellent agreement (A3 – 0.927) to poor agreement (C1 – 0.123) (Table 5). The best agreement was seen in A3, H3 (0.925), and E1 (0.918). Simple linear regression tests of the *y*-intercept = 0 and the slope = 1 were conducted and all methods except for A3, E1, F2, G3, and H3 showed a significant difference in at least one of the two tests. Therefore, any linear agreement seen in these methods would be on the 1 to 1 scale that we would want our eSA method to have with aSA. From these methods, it was determined that H3, A3, G3, E1, and F2 were the five best estimation methods of aSA (Table 6). Calculated prediction 95% CIs were narrowest for H3 method at ±45.9 mm^2^ with A3 next best at ±47.8 mm^2^. Representative linear regression models for H3, A3, G3, and E1 are depicted in Figures 5–8.

**Table 2:** **Table reporting aSA and five best eSA and the dimensions of the tooth dies used to calculate eSA for each tooth in the study**.

Die #	Base axes (mm)		Top axes (mm)		Height (mm)	aSA (mm^2^)	eSA H3 (mm^2^)	eSA A3 (mm^2^)	eSA G3 (mm^2^)	eSA E1 (mm^2^)	eSA F1 (mm^2^)
	Major	Minor	FP	MD							
1	12.53	7.42	1.09	4.88	11.43	210.59	253.90	247.29	196.53	256.54	348.32
2	10.06	5.75	3.02	3.14	8.92	156.85	167.25	153.16	121.91	161.82	206.12
3	9.38	6.04	1.00	4.18	9.25	162.99	162.44	153.91	121.93	152.81	218.38
4	11.31	6.82	1.94	1.87	20.46	336.90	363.85	379.36	302.14	377.11	437.46
5	8.37	5.48	1.57	1.55	9.65	162.42	133.90	141.60	112.18	138.29	161.37
6	9.65	5.07	4.19	3.51	7.32	115.84	145.45	119.20	95.14	132.89	173.76
7	7.61	5.25	0.86	1.73	8.02	119.26	103.92	110.82	87.59	106.11	127.67
8	10.65	6.36	4.40	3.28	7.07	147.06	154.62	138.85	110.03	148.07	182.90
9	9.36	5.80	2.96	3.45	12.96	242.90	232.39	204.22	162.30	202.59	276.82
10	9.53	5.58	1.65	1.02	9.78	152.33	145.13	157.63	125.44	162.86	177.57
11	9.92	5.96	4.93	3.54	9.18	180.35	194.16	157.93	125.44	162.59	215.32
12	11.13	7.16	2.33	1.43	9.45	151.42	178.42	191.00	151.19	191.73	211.17
13	11.76	6.78	1.85	2.19	11.85	207.95	224.00	234.64	186.85	244.37	283.80
14	9.29	6.82	4.47	3.94	11.63	204.02	238.75	198.00	156.24	182.75	260.19
15	9.53	7.34	4.12	2.28	9.94	158.73	190.21	181.89	143.29	165.01	200.36
16	8.32	6.09	0.34	3.33	8.88	115.96	135.63	137.82	108.73	128.16	177.50
17	7.53	6.23	0.70	2.65	7.20	98.95	105.54	109.69	86.28	96.10	127.08
18	10.39	6.41	3.90	1.72	13.89	267.87	256.61	243.17	193.26	242.03	279.11
19	10.62	5.76	4.61	1.01	8.37	133.53	156.72	151.10	120.45	165.35	177.11
20	7.72	5.46	2.38	1.90	13.99	183.29	191.55	189.28	149.65	175.99	218.76
21	7.74	4.97	1.03	0.91	12.75	160.53	149.80	166.76	132.37	162.00	180.00
22	8.30	5.72	4.14	3.63	13.31	232.63	241.78	192.74	152.51	181.77	263.58
23	7.73	5.32	1.49	2.85	9.95	159.49	143.53	136.40	107.88	129.61	176.64
24	10.64	6.98	2.09	1.84	16.71	301.03	292.40	304.04	241.06	293.09	341.40
25	10.58	5.94	3.88	3.18	13.80	230.02	271.16	237.14	189.40	245.62	316.75
26	11.42	6.55	7.16	4.57	5.86	136.18	170.26	130.25	103.11	146.77	186.88
27	8.69	5.37	1.30	1.24	13.97	185.54	188.68	202.18	160.77	199.70	226.73
28	9.48	5.70	2.14	1.49	8.76	135.90	139.35	144.10	114.45	148.32	167.65
29	9.15	6.79	1.93	1.67	15.38	204.74	242.76	253.07	199.72	230.63	271.21
30	9.13	7.03	1.34	2.38	15.75	230.20	250.51	262.61	207.03	235.17	295.67
31	11.06	7.44	1.48	0.81	11.28	246.80	196.32	224.83	177.39	218.25	231.53
32	8.61	5.92	1.22	2.74	5.81	86.58	93.26	98.95	78.07	97.80	121.95

**Table 3:** **Akaike information criteria (AIC) and corresponding Chi-square test results for the eSA methods**.

Method	AIC	Pr (Chi)
H3	196.61	3.97E−16
B3	196.78	4.32E−16
G3	199.22	1.49E−15
A3	199.34	1.58E−15
C3	199.93	2.14E−15
A1	204.11	1.78E−14
E1	204.11	1.78E−14
D1	204.12	1.80E−14
C1	204.12	1.80E−14
F1	205.35	3.37E−14
B1	205.35	3.37E−14
E2	209.15	2.31E−13
A2	209.15	2.31E−13
D2	210.20	3.96E−13
C2	210.20	3.96E−13
B2	216.50	9.83E−12
F2	216.50	9.83E−12

**Table 4:** **Pearson correlation coefficients (PCC) for eSA methods**.

Method	PCC
H3	0.94
B3	0.93
A3	0.93
G3	0.93
C3	0.93
A1	0.92
C1	0.92
D1	0.92
E1	0.92
B1	0.91
F1	0.91
A2	0.90
E2	0.90
C2	0.90
D2	0.90
B2	0.88
F2	0.88

**Table 5:** **Intraclass correlation coefficients (ICC) for eSA methods**.

Method	ICC (2,1)	ICC 95% CI
A3	0.93	0.86–0.96
H3	0.93	0.84–0.96
E1	0.92	0.84–0.96
B2	0.82	0.54–0.92
F2	0.81	0.42–0.93
G3	0.76	−0.05 to 0.93
F1	0.73	−0.05 to 0.92
A2	0.71	−0.07 to 0.91
A1	0.68	−0.08 to 0.90
B3	0.64	−0.08 to 0.89
D2	0.61	−0.09 to 0.87
B1	0.42	−0.07 to 0.78
E2	0.42	−0.07 to 0.78
C2	0.35	−0.08 to 0.72
C3	0.20	−0.05 to 0.55
D1	0.20	−0.06 to 0.55
C1	0.12	−0.05 to 0.41

**Table 6:** **Fitted regression estimates for eSA methods**.

Method	Slope estimate (mm^2^/mm^2^)	*Y* -intercept estimate (mm^2^)
A1	0.70 (0.58, 0.81)	18.81 (-9.00, 46.61)
A2	1.06 (0.87, 1.25)	30.37 (1.80, 58.94)
A3*	0.87 (0.74, 1.00)	19.83 (-5.54, 45.19)
B1	0.59 (0.49, 0.68)	13.16 (-16.19, 42.51)
B2	0.74 (0.59, 0.89)	31.58 (-1.13, 64.29)
B3	0.70 (0.60, 0.80)	10.76 (-14.71, 36.22)
C1	0.32 (0.26, 0.37)	17.73 (-10.25, 45.71)
C2	0.50 (0.41, 0.59)	26.36 (-3.48, 56.20)
C3	0.40 (0.34, 0.46)	16.45 (-9.67, 42.57)
D1	0.40 (0.34, 0.47)	17.73 (-10.25, 45.71)
D2	0.63 (0.52, 0.75)	26.36 (-3.48, 56.20)
E1*	0.89 (0.74, 1.03)	18.81 (-9.00, 46.61)
E2	1.35 (1.11, 1.59)	30.37 (1.80, 58.94)
F1	0.75 (0.62, 0.87)	13.16 (-16.19, 42.51)
F2*	0.94 (0.75, 1.14)	31.58 (-1.13, 64.29)
G3*	1.10 (0.93, 1.26)	20.31 (-4.93, 45.55)
H3*	0.89 (0.76, 1.02)	11.58 (-13.70, 36.85)

*Slope should equal 1 mm^2^/mm^2^ and *y*-intercept should equal 0 mm^2^ for perfect agreement*.

*Both criteria of 95% CIs contain 1 mm^2^/mm^2^ for slope and 0 mm^2^ for *y*-intercept met.

## Discussion

Mathematical modeling of tooth crown SA is an uncommon research approach. A thorough review of the literature reveals only three prior human prosthodontic studies utilizing mathematical models (12–14). The first study evaluated the influence of the interaction between vertical crown height and crown inclination on crown SA by utilizing a mathematical model based on a right pyramidal frustum (12). The right pyramidal frustum was chosen by the authors because of its resemblance to the human molar. In a second study conducted by the same author, the same mathematical model of a right pyramidal frustum was used to investigate the influence of axial grooves on the overall SA (13). A third study utilized a similar study design to compare SA calculations based on the right conical frustum (truncated cone) to those calculations based on the right pyramidal frustum (14). Although the authors stated that SA calculations made using the right conical frustum were closer to clinically calculated crown preparation SA, they were also ambiguous about which method was the most useful (14).

The right pyramidal frustum and right conical frustum utilized in these previous studies would be analogous to the B1, B2, B3 and the F1, F2 models, respectively, utilized in the present study. The right pyramidal frustum (B1, B2, B3) models were among the methods with the least degree of agreement between eSA and aSA. Methods utilizing a right circular frustum (F1 and F2) provided better agreement between eSA and aSA than the methods utilizing the right pyramidal frustum. The morphology of the dog canine tooth is starkly different than the human molar. The dog canine tooth has a base that is typically elliptical; although it has been the authors’ experience that some canine teeth will have a more circular base. The human molar is naturally more cuboidal. However, preparation of the tooth to receive a full coverage crown often results in a morphology that more closely resembles the pyramidal frustum. Therefore, the results of the present study using right pyramidal frustum models and indicating comparatively poor correlation between eSA and aSA were expected.

To the authors’ knowledge, the present study is the first that has attempted to systematically correlate eSA made with mathematical modeling of various geometric shapes with aSA calculations acquired through laser-scanning and 3D computer models. Additionally, in the present study, we utilized physical dimensions acquired from actual clinical cases to make our calculations, rather than theoretical dimensions that simulate crown preparations as has been carried out in previous studies.

The unaltered canine tooth has a complicated geometric shape, which may be characterized as an eccentric elliptical cone with a dynamic curvature of the distal surface. This distal curvature can be quite extreme, particularly in the event of abrasion of the distal surface, which happens to be one of the more common indications for full coverage crown therapy. Perhaps the most common indication for full veneer crown therapy of the canine tooth in dogs is to add additional protection to the tooth after treatment (e.g., root canal therapy) for a traumatic dentoalveolar injury. Traumatic dentoalveolar injuries affect just over one in four dogs, the most common injury being a crown fracture (15). Crown fractures can significantly alter the already complex shape of the canine tooth. In the event of iatrogenic crown shortening or in the occurrence of transverse or short oblique crown fractures, the crown shape is altered from an elliptical cone to an elliptical frustum. Thus, the natural tooth and any event that alters the shape of the tooth present a complex task of estimating tooth SA. Therefore, the basic geometric shapes chosen for the calculation of eSA varies from those that closely resemble the shape of the dog’s canine tooth to those that have very little resemblance to the canine tooth.

Hypothetically, utilizing a geometric shape that is a good representation of the actual tooth should be a more accurate method to estimate SA. Method H3 utilized the base geometric shape of a right elliptical frustum, which resembles a tooth that has been shortened in height by either a traumatic dentoalveolar injury or by iatrogenic intervention. Based on the data, method H3 was the most accurate method for estimating crown SA. However, method H3 requires the use of laser scans and a 3D CAD software program. As a result, this method would not likely be feasible as a chair-side method and would be recommended as the most appropriate method for estimating crown SA in a research setting.

Method G3 was found to have comparable accuracy to H3. Method G3 was based on the basic geometric shape of a right elliptical cone, which most accurately represents the shape of the intact canine tooth. Like the H3 method discussed above, utilization of the G3 method also requires the use of a computer software program and a more sophisticated equation. Thus, the G3 method would also be more useful for research applications rather than chair-side applications.

It is interesting to note that the two basic geometric shapes, as utilized in methods H3 and G3, that provide the most accurate estimations of crown SA were the right elliptical cone and the right elliptical frustum. These shapes best represent the actual geometry of the intact canine tooth and the fractured (transverse) canine tooth, respectively. The test subjects in this study consisted of both intact canine teeth and canine teeth with transverse crown fractures. Therefore, the finding that these two methods provided similar results is not surprising.

Methods A3, E1, and F2 represented the basic geometric shapes of pyramidal cone, circular cone, and circular frustum, respectively. These geometric shapes were included in the study because, on an elementary level, these geometric shapes resemble the shape of the tooth. As noted previously, these same geometric shapes have also been used as mathematical models to determine crown SA for human molar teeth (12–14). As anticipated, they were not as accurate for estimating canine tooth crown SA as the more elliptical geometric shapes. However, the difference in accuracy between the five most accurate methods was small. Additionally, there is a decided advantage to A3, E1, and F2 in that they require relatively simple mathematical equations. Thus, they are much more easily implementable for chair-side use. In particular, the 95% CI and the linear regression models reveal method H3 and E1 to be very similar in accuracy. The SA calculation on E1 would be considered the simplest equation and could be very easily performed chair-side with only two measurements (major base diameter and crown height); whereas methods A3 and F2 each require three measurements.

The authors acknowledge a limitation with the findings of this study. The 95% CI for the most accurate methods to estimate SA was within ±52 mm^2^. Given the aSA of the subject teeth, this CI is relatively large, and equates to possibly sizeable variability between aSA and eSA. In our previous work, we have shown a trend toward a positive association between aSA acquired with 3D laser scans and clinical crown retention (5). Additionally, *ex vivo* studies have shown a significant correlation between abutment SA and the force required to dislodge a full veneer crown (4). We propose that estimation of crown SA may prove to be a clinically useful technique to predict the risk of crown dislodgement. However, the question remains whether the relatively large variability between aSA and eSA would be clinically significant when applied to risk of crown dislodgement assessment. Clinical application of crown SA estimation should be pursued in order to determine if the measurement could be used as a predictor of crown dislodgement risk.

In conclusion, eSA calculations utilizing methods H3 (right elliptical frustum), G3 (right elliptical cone), A3 (right pyramidal cone), E1 (right circular cone), and F2 (right circular frustum) showed significant linear association and agreement with aSA. Methods H3 and G3 require software programs for calculation and, thus, may be more appropriate for research applications. A3, E1, and F2 can all be calculated relatively easily using measurements acquired chair-side. However, we recommend the use of method E1 for chair-side estimations of crown SA of dog teeth due to its high linear association with aSA and to the comparatively elementary nature of the required calculation.

## Conflict of Interest Statement

The authors declare that the research was conducted in the absence of any commercial or financial relationships that could be construed as a potential conflict of interest. The Associate Editor Frank J. M. Verstraete declares that, despite having collaborated on a publication in the last 2 years with author Jason W. Soukup, the review process was handled objectively and no conflict of interest exists.
